# Charged Amino Acid Substitutions Affect Conformation of Neuroglobin and Cytochrome *c* Heme Groups

**DOI:** 10.3390/cimb46040211

**Published:** 2024-04-14

**Authors:** Marina A. Semenova, Zhanna V. Bochkova, Olga M. Smirnova, Georgy V. Maksimov, Mikhail P. Kirpichnikov, Dmitry A. Dolgikh, Nadezda A. Brazhe, Rita V. Chertkova

**Affiliations:** 1 Shemyakin-Ovchinnikov Institute of Bioorganic Chemistry, Russian Academy of Sciences, Miklukho-Maklaya St. 16/10, 117997 Moscow, Russia; marinaapbch@mail.ru (M.A.S.); zh.bo4kova@yandex.ru (Z.V.B.); olyasmirnova00@mail.ru (O.M.S.); kirpichnikov@inbox.ru (M.P.K.); dolgikh@nmr.ru (D.A.D.); 2 Biophysics Department, Biological Faculty, Lomonosov Moscow State University, Leninskie Gory, 1/12, 119899 Moscow, Russia; gmaksimov@mail.ru; 3 Biology Department, Lomonosov Moscow State University, Leninskie Gory, 1/12, 119899 Moscow, Russia

**Keywords:** neuroprotection, cytochrome *c*, neuroglobin, heme, heme proteins, resonance Raman spectroscopy, surface-enhanced Raman spectroscopy

## Abstract

Neuroglobin (Ngb) is a cytosolic heme protein that plays an important role in protecting cells from apoptosis through interaction with oxidized cytochrome *c* (Cyt *c*) released from mitochondria. The interaction of reduced Ngb and oxidized Cyt *c* is accompanied by electron transfer between them and the reduction in Cyt *c*. Despite the growing number of studies on Ngb, the mechanism of interaction between Ngb and Cyt *c* is still unclear. Using Raman spectroscopy, we studied the effect of charged amino acid substitutions in Ngb and Cyt *c* on the conformation of their hemes. It has been shown that Ngb mutants E60K, K67E, K95E and E60K/E87K demonstrate changed heme conformations with the lower probability of the heme planar conformation compared to wild-type Ngb. Moreover, oxidized Cyt *c* mutants K25E, K72E and K25E/K72E demonstrate the decrease in the probability of methyl-radicals vibrations, indicating the higher rigidity of the protein microenvironment. It is possible that these changes can affect electron transfer between Ngb and Cyt *c*.

## 1. Introduction

Neuroglobin (Ngb) is a six-coordinate heme protein from the globin family predominantly expressed in nervous tissue [1]. Ngb consists of eight α-helices (A-H) and displays typical three over three globin fold. Ngb contains one heme group, and in the absence of exogenous ligands, the axial coordination positions are occupied by His96 (proximal histidine) and His64 (distal histidine) [2,3]. Two cysteine residues (Cys46 and Cys55) form an intramolecular disulfide bridge that modulates Ngb ligand binding and redox properties [3,4,5]. Ngb can bind small gaseous ligands (O_2_, CO, NO) [6,7] and also exhibit nitrite reductase activity [8,9].

Significant amounts of evidence support a physiological role of Ngb in the promotion of neuronal survival in conditions such as Alzheimer’s and Huntington’s diseases, oxidative stress, stroke, ischemia, hypoxia, retinal degeneration, etc. [10,11,12,13,14,15,16,17,18,19,20]. Ngb-mediated neuroprotection is proposed to be due to Ngb involvement in various biochemical cascades of the cell [10,12,14,15,20]. Ngb has the ability to detoxify reactive oxygen and nitrogen species [11,13]. Furthermore, protein–protein interactions between Ngb and voltage-dependent anion channels, the α-subunit of heterotrimeric G-protein, and mitochondrial cytochrome *c* (Cyt *c*) can also play crucial roles in Ngb-mediated promotion of neuronal survival [14,20,21,22].

Thus, at least one of the Ngb-mediated neuroprotection mechanisms may be due to interactions between Ngb and Cyt *c* [21,22,23,24,25,26]. Cyt *c* is a heme protein with the main function of electron transfer (ET) in the respiratory chain. However, Cyt *c* is a multifunctional protein involved in a variety of cell processes. One of the most important Cyt *c* activities besides ET is the initiation of apoptosis via interaction with apoptotic protease activating factor-1 (Apaf-1) [27]. Intermolecular ET from ferrous Ngb to ferric Cyt *c* can prevent Cyt *c*-dependent (intrinsic) apoptotic pathway initiation because ferrous Cyt *c* cannot participate in the reaction with Apaf-1; thus, it lacks its apoptosis initiation capacity [28].

The ET presumably occurs within a transient Ngb/Cyt *c* complex. However, the structure of the complex is unknown, whereas models of the Ngb/Cyt *c* complex have been obtained by bioinformatics methods [29,30,31,32,33]. The findings from the binding surfaces modeling and the identification of key amino acid residues on the surfaces of Ngb and Cyt *c* demonstrated that electrostatic interactions are crucial for the transient Ngb-Cyt *c* complex formation [29,30,31,32,33], although hydrophobic interactions and hydrogen bonds are also likely to contribute to the Ngb-Cyt *c* complex stabilization [32,33]. The Ngb amino acid residues involved in interactions with Cyt *c* belong to E and F α-helices, while most of the Cyt *c* interaction residues belong to either a Cyt *c* universal binding site or Ω-loop (70–85) [32,33]. Glu60 and Glu87 (Ngb) presumably interact with Cyt *c* residues Lys72 and Lys25 [29,30,31]. Notably, Cyt *c* Lys72 is considered to be the key residue for the Cyt *c* interaction with Apaf-1, whereas Lys25 is also involved [34]. Ngb Lys67 and Lys95 may interact with residues Ile81 and Gln16 of Cyt *c* [29,32,33]. However, there is a lack of empirical evidence in support of those models. Ngb and Cyt *c* are both heme proteins, and the ET between them depends on two processes: the interaction of proteins and the optimization of hemes’ conformation to ensure the tunneling of the electron between hemes. Amino acid substitutions can cause alterations of heme properties, which in turn can affect the ET between these proteins. Hence, it is necessary to study the effect of site-specific mutations in the putative interaction surface of Ngb and Cyt *c* on the conformation of their hemes. Resonance Raman (RRS) spectroscopy is a relevant method that is highly sensitive to redox and conformational changes of hemes in hemoproteins. A better understanding of heme properties changes of isolated mutant variants of Ngb and Cyt *c* is crucial as a basis for the further studies of molecular interactions between Ngb and Cyt *c*, which will consequently lead to rational design of new therapeutic agents for inhibiting neuronal cell death under various pathophysiological conditions such as ischemia and hypoxia.

In previous works, we developed a wild-type (WT) and mutant human Ngb biosynthesis system in *Escherichia coli* [35,36]. We described the characteristic RRS and surface-enhanced Raman spectra (SERS) of ferrous and ferric Ngb WT, Cyt *c* WT and a number of mutant Cyt *c* variants in detail [36,37].

The aim of this study was to investigate how substitutions of charged amino acids on the surface of Ngb and Cyt *c* affect proteins’ heme conformation. Therefore, we studied the conformations of hemes of Cyt *c* mutants (K25E, K72E, K25E/K72E) with substitutions in the putative interaction surface with Ngb and hemes of Ngb mutants (E60K, E87K, K67E, K95E, E60K/E87K) with substitutions in the putative interaction surface with Cyt *c*, using Raman spectroscopy methods.

## 2. Materials and Methods

### 2.1. Reagents

Components for the culture media and buffer solutions for chromatography and electrophoresis, ampicillin (AppliChem, Darmstadt, Germany), *Pfu*-DNA polymerase (Fermentas, Pabrade, Lithuania), and *Mal* I restrictase (SibEnzyme, Novosibirsk, Russia) were used in this study. Distilled water was additionally purified on a Milli-Q system (Millipore, Burlington, MA, USA). We used sodium dithionite (DIAM, Frankfurt, Germany) for the proteins reduction.

### 2.2. Construction of the Mutant Genes of Ngb and Cyt c

The mutagenesis primers for the introduction of amino acid substitutions in the putative interaction surface of Ngb and Cyt *c* can be found in Table 1.

The mutations were introduced in the pET17-Ngb and pBP(CYC1/CYC3) plasmid sequence by site-directed mutagenesis according to the QuikChange^TM^ Mutagenesis Kit method (Stratagene, La Jolla, CA, USA) as described previously [35]. The reaction mixture (50 µL) contained 10 ng of matrix DNA (the pET17-Ngb or pBP(CYC1/CYC3), oligonucleotide primers (125 ng) with mutations E60K, E87K, K67E, K95E and E60K/E87K for the Ngb gene, K25E, K72E, and K25E/K72E for the Cyt *c* gene, four deoxynucleoside triphosphates (10 nmol of each), and *Pfu* polymerase (2.5 U). Twenty cycles of the amplification reaction were performed according to the following scheme: denaturation of the matrix DNA at 95 °C for 30 s, annealing at 55 °C for 60 s, and elongation at 72 °C for 10 min. When the reaction was completed, the *Mal* I restrictase (10 U) was added, and the reaction mixture was incubated for 60 min at 37 °C. Furthermore, aliquots of the prepared mixture were used for transformation of the *E. coli* JM-109 competent cells according to the standard procedure. The production of mutant DNA during mutagenesis was analyzed by electrophoresis in 1% agarose gel.

### 2.3. Expression of Ngb and Cyt c, Protein Isolation, and Purification

The expression of the wild-type and mutant genes was performed in the *E. coli* JM-109 in an SB liquid-nutrient medium with ampicillin (the final concentration was 150 µg/mL) without the addition of the inductor at 37 °C under intensive stirring for 24 h for Cyt *c* [38] and in the *E. coli* SHuffle T7 in an TB liquid-nutrient medium with ampicillin (the final concentration was 150 µg/mL) without the addition of the inductor at 28 °C under intensive stirring for 24 h for Ngb [36]. The plasmid vector pBP(CYC1)/(CYC3) was developed for co-expression in *E. coli* of the yeast cytochrome *c* and yeast heme ligase (CYC3) genes [39], which are under the control of successively located *lac* and *trc* promoters. The pBP(CYC1)/(CYC3) design allows one to obtain a high level of expression of heme-containing cytochrome *c* in *E. coli*. A modified version of this system was obtained for the expression of the horse Cyt *c* gene in bacterial cells, and we used it in the present work.

Afterward, the growth cells were homogenized by forcing through a French press (Spectronic Instruments, Inc., Irvine, CA, USA) at high pressure with subsequent centrifugation at 95,000× *g* for 30 min.

The isolation and purification of the target proteins were performed on an “AKTA FPLC” liquid chromatographic system (GE HealthCare, Chikago, IL, USA) according to the previously elaborated schemes [36,40]. The degree of purification of proteins in the resulting fractions was determined by electrophoresis in 12% Tris-Tricine SDS-PAGE [41]; see Figure S1 in the Supplementary Materials. The Ngb fractions with the purity of approximately ≥90–95% were dialyzed three times against 10 mM ammonium carbonate buffer (pH 7.9), and lyophilized on an ALPHA I-5 device. The Cyt *c* resulting fractions were oxidized by treating with potassium ferricyanide added at the equimolar concentration before three times dialysis against 10 mM ammonium carbonate buffer (pH 7.9), and then they were lyophilized on an ALPHA I-5 device. For Ngb fractions, no additional oxidation was required. The Ngb and Cyt *c* concentrations were determined by absorbance measured at the Soret region (Figure S2) on a Cary-50 spectrophotometer (Varian, Cary, NC, USA).

### 2.4. Resonance Raman and Surface-Enhanced Raman Spectroscopy of Ngb and Cyt c

The RRS and SERS spectra of WT and mutant Ngb and Cyt *c* molecules were recorded using confocal Raman spectrometer NTEGRA Spectra (NT-MDT, Zelenograd, Russia) coupled to the inverted IX-71 microscope (Olympus, Tokyo, Japan), objective ×20 NA 0.45. To record Raman or SERS spectra, we used laser excitation at 532 nm wavelength with the power of 1 mW per the registration spot with the diameter of approx. 800 nm. The spectrum accumulation time was 1 s. All measurements were performed in 30 mM PBS containing 7 mM EDTA, pH 6.5, 22 °C. For SERS measurements of oxidized protein samples, we used silver (Ag) nanostructured surfaces prepared as described previously [37]. Then, 15 µL of protein (Ngb or Cyt *c*) solution was placed on the glass bottom of a Petri dish and covered with nanostructures so that the side with a silver nanostructural surface was turned toward the laser ray and the objective. We used solutions of ferric and ferrous WT and mutant proteins at the concentration of 1 mM. The reduction in WT and mutant proteins was performed with sodium dithionite (Sigma, St. Louis, MO, USA). A small amount of sodium dithionite powder was added into the experimental probe with protein solution of 15 µL volume before spectrum recording. In all cases, the number of independent measurements was 3–5. SERS and Raman spectra were analyzed with the open-source software Pyraman version v0.3.4-beta, which is available at https://github.com/abrazhe/pyraman/releases/tag/v0.3.4-beta, accessed date 13 April 2024. After the baseline subtraction, the intensities of peaks with certain maximum positions were defined and further used in calculations of peak intensities ratios characterizing heme conformation. The detailed assignments of these peaks and their ratios is discussed in the Section 3.

## 3. Results

**Figure 1:**
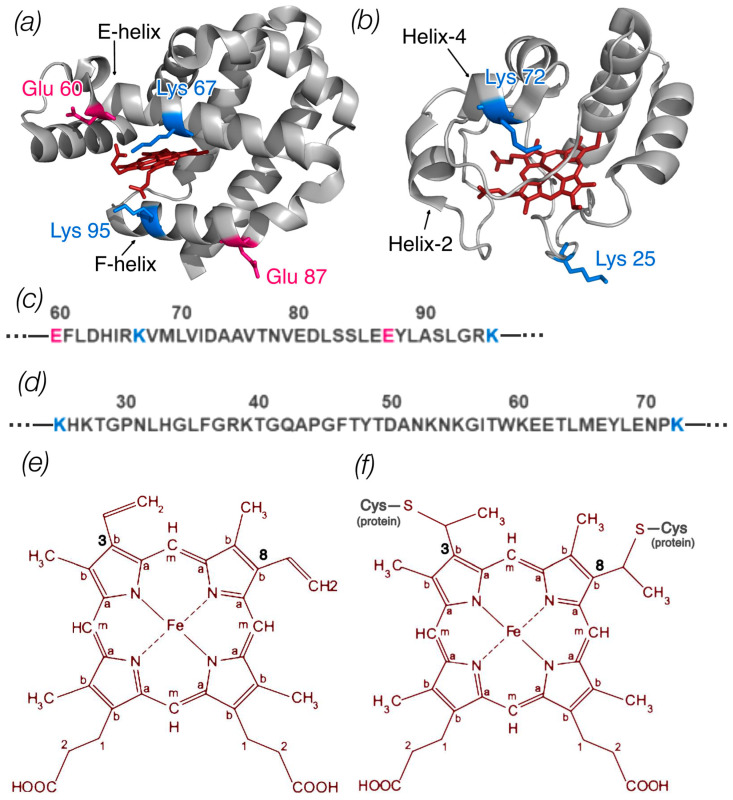
(**a**) Ngb structure (ID PDB: 4MPM) and (**b**) Cyt *c* structure (ID PDB: 1HRC); the image was generated with the PyMOL program. The amino acid residues of Ngb and Cyt *c* that were changed in mutant variants are shown coded in colors according to amino acid type. Parts of the protein sequence of Ngb (**c**) and Cyt *c* (**d**) with these amino acids are also shown. (**e**) Schematic representation of the heme *b* structure (Ngb) with labeled carbon atom types: C_a_ in the pyrrole ring is connected to the carbon of the methine bridge, C_b_ in the pyrrole ring forms a bond with the side radicals, and C_m_ is the carbon of the methine bridge. At positions 3 and 8, heme b has two vinyl groups. (**f**) Schematic representation of the heme *c* structure (Cyt *c*) with labeled types of carbon atoms. At positions 3 and 8, heme *c* is bound to the protein via two cysteine residues.

Based on literature data [29,30,31,32,33], amino acid substitutions were made in the putative region of the protein involved in the formation of the Ngb complex (E60K, E87K, K67E, K95E, E60K/E87K) [35] and Cyt *c* (K25E, K72E, K25E/K72E) (Figure 1). These mutations can change the local charge of the protein globule and could disrupt the electrostatic interactions between protein molecules. Furthermore, Glu60, Lys67, and Lys95 Ngb are located in close proximity to the heme [3]. In the case of Cyt *c*, Lys72 belongs to the disordered red Ω-loop [27]. Therefore, there is a possibility of protein conformation changes caused by amino acid substitutions, affecting the heme conformation as well. It is likely that such changes if present can be crucial for the stability of Ngb/Cyt *c* complexes and the efficiency of ET.

### 3.1. Conformation of Heme and Its Local Protein Environment in Ngb and Cyt c Are Affected by the Mutations in the Putative Interaction Surface

We studied conformational heme properties and its environmental lability in WT and mutant forms of ferrous and ferric Ngb and Cyt *c* molecules in the phosphate buffer solution. Resonance Raman scattering with laser excitation at 532 nm allows to investigate conformational heme properties and heme protein surroundings. Raman peaks correspond to certain modes of normal group vibrations of heme atoms, and peak intensities depend on the probability of such vibrations so that the Raman spectrum can be used to evaluate the relative content of atom groups with certain vibrational modes.

However, the spectra of heme-containing proteins in the oxidized state are known for low intensity compared to the reduced state [42,43]. In addition, we found out that Raman scattering intensity of oxidized Ngb (WT) and its forms was lower than that of the Cyt *c* (WT) forms. In order to enhance the Raman signal of oxidized Ngb and Cyt *c* wild-type and mutant forms, we used surface-enhanced Raman spectroscopy (SERS) based on the many-fold increase in the Raman scattering intensity of the molecules located in the close vicinity of plasmonic nanostructured surfaces.

### 3.2. Spectral Heme Characteristics of Ngb (WT), Cyt c (WT) and Their Mutant Forms in Oxidized and Reduced State Obtained by SERS and Raman Scattering, Respectively

**Figure 2:**
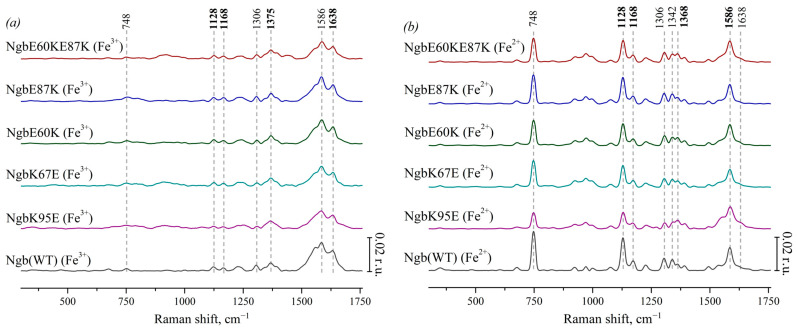
(**a**) SERS spectra of oxidized Ngb (WT) (black line) and its mutants with amino acid substitutions: Ngb K95E (purple), Ngb K67E (dark cyan), Ngb E60K (dark green), Ngb E87K (royal blue), and Ngb E60K/E87K (wine). (**b**) Resonance Raman spectra of reduced WT Ngb and mutant forms (the same color coding). All spectra are normalized to the sum of their intensities in the whole spectrum range. Vertical scale bars correspond to 0.02 r.u. For better representation, spectra are shifted vertically one over another. X axes correspond to Raman shift, which is shown in cm^−1^. Dotted lines show the maximum positions of the most intensive peaks and peaks used in the analysis of conformational changes.

Characteristic peaks of heme components were identified, and their physical interpretation was determined due to the literature data. Figure 2a presents the SERS spectra of Ngb (WT) and its mutants with substitutions responsible for electrostatic interaction with Cyt *c*: Ngb (K95E) (purple), Ngb (K67E) (dark cyan), Ngb (E60K) (dark green), Ngb (E87K) (royal blue), and Ngb (E60K/E87K) (wine). These SERS spectra demonstrate typical peaks of the SERS spectra of heme-containing proteins [44]: 748 cm^−1^ (the mode is associated with vibrations of all bonds in the porphyrin ring), 1128 cm^−1^ (originates from the CH_3_-side radical’s vibrations), 1168 cm^−1^ (asymmetric vibrations of the pyrrole half-rings), and 1375 cm^−1^ (symmetrical vibrations of pyrrole half-rings). All SERS spectra of Ngb forms contain a peak at 1306 cm^−1^ which is the characteristic feature of the heme *b* type. High-frequency peaks with maxima at 1586 and 1638 cm^−1^ originate from the normal group vibrations of methine bridges and are known to be sensitive to the out-of-plane distortions of heme in various hemoproteins, increasing their intensities under the planar heme conformations and decreasing intensities under dome or ruffled heme conformations [44].

Figure 2b shows the corresponding resonance Raman spectra of Ngb and its mutants with amino acid substitutions in the reduced state. The peak with a maximum at 1342 cm^−1^ is clearly visible on the spectra. It is known to be another characteristic peak of heme *b* that is intensive in the reduced state of the heme [42,43], which makes it possible to distinguish the spectra of Ngb and its mutants from hemoproteins with heme *c*. Under the reduction in Ngb, we observed the following spectral changes: the peak with the maximum position at 1375 cm^−1^ (symmetric vibrations of pyrrole half-rings) in the spectra of oxidized Ngb and its mutants shifts to the position at 1368 cm^−1^ in the spectra of the reduced molecules. Both peaks at 1375 and 1368 cm^−1^ are from the same mode (ν_4_), and this shift is well known for heme-containing proteins. Also, under Ngb reduction, the peak at 1638 cm^−1^ (vibrations of the methine bridges) shifts to the lower-frequency region and merges with the peak at 1586 cm^−1^. The peaks with maximum positions at 748, 1128, 1306, 1342, and 1586 cm^−1^ show the significant increase in the intensity under reduction in Ngb. Such an effect is also known for reduced *c*- and *b*-types cytochromes [42,43]. We did not observe any frequency shifts of the characteristic peaks on the RRS and SERS spectra of Ngb mutant forms comparing to the positions of these peaks on the spectra of the Ngb WT. This is consistent with the fact that the introduced amino acid substitutions did not directly lead to the restructuring of the heme ring.

**Figure 3:**
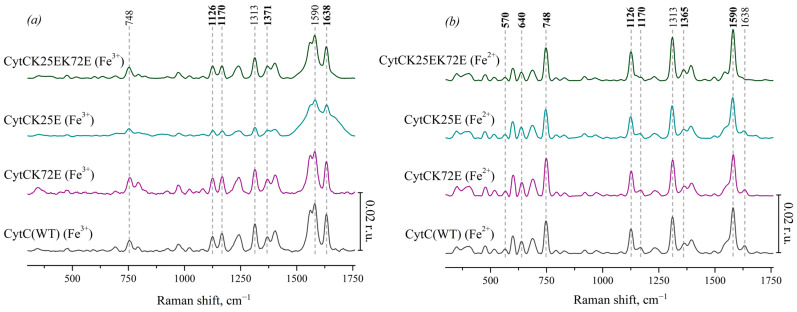
(**a**) SERS spectra of the oxidized Cyt *c* (WT) (black line) and the mutant forms with amino acid substitutions: Cyt *c* K72E (purple), Cyt *c* K25E (dark cyan), Cyt *c* K25E/K72E (dark green). (**b**) Resonance Raman spectra of the reduced Cyt *c* (WT) and the mutants. All spectra are normalized to the sum of their intensities in the whole spectral range. Vertical scale bars correspond to 0.02 r.u. For better representation, spectra are shifted vertically one over another. X axes correspond to the Raman shift, cm^−1^. Dotted lines show the maximum positions of the most intensive peaks and peaks used for the analysis of heme conformational changes.

We also used the SERS method to obtain Raman spectra of oxidized Cyt *c* and its mutant forms: Cyt *c* (K25E), Cyt *c* (K72E) and Cyt *c* (K25E/K72E) (Figure 3a). These spectra show the same bands that are presented on the SERS spectra of oxidized Ngb: 748 cm^−1^ (vibrations of all pyrrole bonds), 1128 cm^−1^ (normal group vibrations of CH_3_-side radicals), 1168 cm^−1^ (asymmetric vibrations of pyrrole half-rings), 1375 cm^−1^ (symmetric vibrations of pyrrole half-rings—pyrrole “breathing”) and high-frequency peaks 1586 and 1638 cm^−1^ (vibrations of methine bridges). At the same time, there is no characteristic peak of heme *b* at 1306 cm^−1^, but there is the peak at 1313 cm^−1^ that is the characteristic feature of heme *c* [37]. The peak positions of SERS spectra of oxidized Cyt *c* mutants do not differ from Cyt *c* WT; however, the SERS spectrum of oxidized Cyt *c* (K25E) demonstrates wider peaks in the region of 1500–1750 cm^−1^ comparing to WT or other mutants of Cyt *c*.

On the RRS spectra of the reduced Cyt *c* and its mutant forms (Figure 3b), as well as on the spectra of the reduced Ngb, the peak maximum at 1371 cm^−1^ shifts to 1365 cm^−1^. The intensities of peaks at 748, 1126, 1313, and 1590 cm^−1^ increase under the reduction in Cyt c as well as in reduced Ngb WT and its mutant forms. In spite of many similarities, there is the remarkable difference of RRS spectra of Cyt *c*(Fe^2+^) and Ngb(Fe^2+^)—low-frequency peaks in the region of 400–750 cm^−1^. Almost all of them originate from the heme c vibrations of various symmetries, and it is known that the peak intensity at 570 cm^−1^ correlates with the degree of non-planar heme distortion (ruffling conformation) [44] and that the peak at 640 cm^−1^ originates from C-S bond vibrations of cysteines 3 and 8 covalently bound to the heme *c* [45]. Their intensities are significantly lower when heme *c* is oxidized.

### 3.3. Study of Conformational Changes of Heme Depending on Its Protein Environment in Ngb and Cyt c WT and Their Mutant Forms

In order to characterize the conformational heme changes in Ngb and Cyt *c* mutants, we analyzed the certain relative peak intensities on the SERS and RRS spectra. The peaks at 1375 and 1368 cm^−1^ on the SERS and RRS spectra of oxidized and reduced Ngb, respectively, represent the same vibrational mode v_4_ and correspond to the symmetric vibrations of pyrrole half-rings (C_a_N, C_a_C_b_ bonds [37]). The Raman shift of this mode depends on the redox state of the iron atom, and its position and intensity do not depend on the out-of-plane deformations of the heme but depend only on Ngb or Cyt *c* concentrations. Hence, intensities of peaks at 1375 and 1368 cm^−1^ are useful for the normalization of the peak intensities dependent on the heme conformation: its out-of-plane changes in heme conformation, heme mobility and the rigidity of the protein microenvironment in the oxidized and reduced hemoproteins, respectively [44].

The following ratios of peak intensities were used to compare Ngb or Cyt *c* heme conformation in WT and mutant forms:

The ratios of peak intensities at 1638 and 1375 cm^−1^ (I_1638_/I_1375_) or 1586 and 1368 cm^−1^ (I_1586_/I_1368_) were used as the indicator of heme out-of-plane distortion in oxidized or reduced Ngb, respectively. The ratios of peak intensities at 1638 and 1371 cm^−1^ (I_1638_/I_1371_) or 1590 and 1365 cm^−1^ (I_1590_/I_1365_) were used as the indicator of heme out-of-plane distortion in oxidized or reduced Cyt *c*, respectively. The decrease in these ratios corresponds to the out-of-plane heme distortion and to the decrease in the probability in the planar heme conformation.

The ratios of the peak intensities at 1168 and 1375 cm^−1^ (I_1168_/I_1375_) or 1168 and 1368 cm^−1^ (I_1168_/I_1368_) were used to estimate the relative intensity of asymmetric vs. symmetric pyrrol rings’ vibrations in oxidized or reduced Ngb, respectively. The ratios of peaks intensities at 1170 and 1371 cm^−1^ (I_1170_/I_1371_) or 1170 and 1365 cm^−1^ (I_1170_/I_1365_) were used to estimate the relative intensity of asymmetric vs. symmetric pyrrol rings’ vibrations in oxidized or reduced Cyt *c*, respectively. These ratios relate to the planar mobility of the pyrrol rings, and the ratios’ decrease corresponds to the decrease in the pyrrols’ mobility.

The ratios of peaks intensities at 1128 and 1375 cm^−1^ (I_1128_/I_1375_) or 1128 and 1368 cm^−1^ (I_1128_/I_1368_) were used for the estimation of the probability of heme methyl-side radicals’ vibrations in oxidized or reduced Ngb, respectively. The ratios of the peak intensities at 1126 and 1371 cm^−1^ (I_1126_/I_1371_) or 1126 and 1365 cm^−1^ (I_1126_/I_1365_) were used for the estimation of the probability of heme methyl-side radicals’ vibrations in oxidized and reduced Cyt *c*, respectively. These ratios relate to the local rigidity of the protein microenvironment of the heme in Ngb and Cyt *c*, and their decrease corresponds to the increase in the heme microenvironment rigidity.

As the additional estimation of the probability of the ruffled heme conformation in Cyt *c* WT and its mutants, we used the ratio I_570_/I_748_; to estimate conformational mobility in the region of the bonds between Cys residues and the heme in Cyt *c*, we used the ratio of peak intensities I_640_/I_1365_.

There are also other peaks in Raman and SERS spectra of Cyt *c* (e.g., 600–604, 680, 1400, etc.) that we did not use for the analysis, since they did not provide additional information to the ratios described above.

#### 3.3.1. Conformational Changes in Ngb Mutants

**Figure 4:**
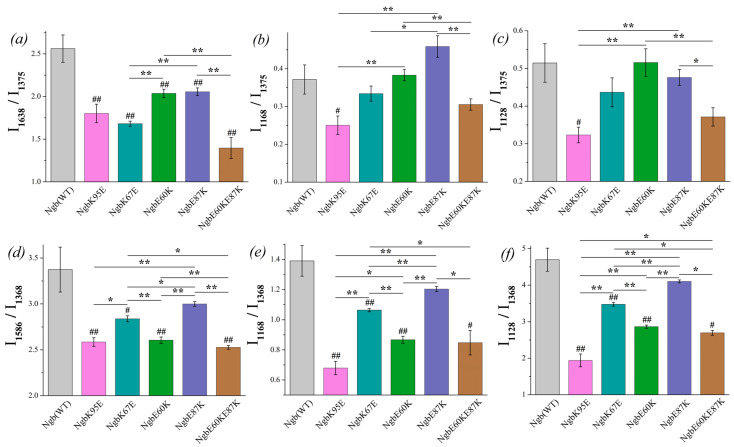
The ratios of the selected peaks intensities calculated from the SERS (**a**–**c**) and RRS spectra (**d**–**f**) of Ngb (WT) (light gray) and its mutants: Ngb K95E (purple), Ngb K67E (dark cyan), Ngb E60K (green), Ngb E87K (navy), and Ngb E60K/E87K (brown). (**a**) I_1638_/I_1375_ ratio and (**d**) I_1586_/I_1368_ ratio correspond to the probability of the planar heme conformation in the oxidized and reduced states, respectively, (**b**) I_1168_/I_1375_ rate and (**e**) I_1168_/I_1368_ rate correspond to the probability of the asymmetric vibrations of pyrrol half-rings, and hence in the conformational mobility of the proteins surrounding the heme molecule (planar mobility of the pyrrols) in the oxidized and reduced state, respectively, (**c**) I_1128_/I_1375_ ratio and (**f**) I_1128_/I_1368_ ratio correspond to the probability of the vibrations of the heme CH_3_-side radicals in the oxidized and reduced state, respectively. Data are presented as mean values and error of the mean, ^#^ *p* < 0.05, ^##^ *p* < 0.01 for Ngb mutants compared to Ngb (WT), * *p* < 0.05, ** *p* < 0.01 for various Ngb mutants compared to each other (nonparametric Mann–Whitney test), *n* = 5.

The ratio I_1638_/I_1375_ shows the significant difference between oxidized Ngb WT and its mutants (Figure 4a, designation # in the diagram). The ratio is decreased for all mutants compared to Ngb WT, indicating that the decrease in the probability of planar heme *b* conformation is supposed to be decreased for the Ngb mutants. The lowest probability of planar heme conformation, and thus the most pronounced heme distortion, are manifested for the double mutant Ngb (E60K/E87K) and then for Ngb (K67E) and Ngb (K95E). In the reduced neuroglobin mutants, the ratio I_1586_/I_1368_ also significantly differs for Ngb WT and all mutants (Figure 4d). Among all of the mutants, Ngb (E60K/E87K) in both reduced and oxidized states demonstrates the most pronounced decrease in the probability of planar heme conformation compared to the wild-type Ngb. Among all oxidized Ngb mutants, Ngb (K95E) is the only oxidized mutant which demonstrates the significant decrease in the probability of the heme methyl radicals vibrations and asymmetric vibrations of pyrrols compared to Ngb WT (Figure 4b,c). There are no significant differences between other mutants and Ngb (WT), although mutant forms Ngb (E87K) and (E60K) show the highest ratios I_1128_/I_1368_ and I_1168_/I_1375_, which are significantly higher than for mutants (Figure 4b,c). In the reduced state, all mutant Ngb forms except for Ngb (E87K) differ from the WT Ngb, demonstrating a significantly lower probability of the planar heme conformation, the decreased probability of the vibrations of methyl-radicals, and the decrease in the in-plane pyrrol rings mobility (Figure 4d–f).

#### 3.3.2. Conformational Changes in Cyt *c* Mutants

**Figure 5:**
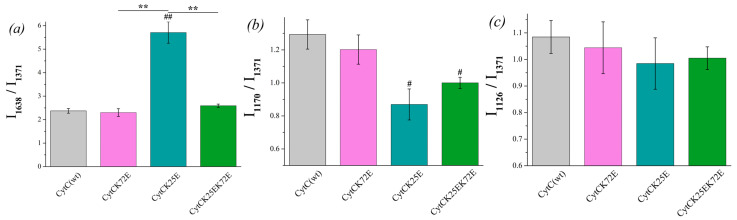
The ratios of the selected peaks’ intensities calculated from the SERS spectra of oxidized Cyt *c* (WT) (light gray) and its mutants: Cyt *c* K72E (purple), Cyt *c* K25E (dark cyan), Cyt *c* K25E/K72E (green). (**a**) I_1638_/I1_371_ ratio corresponds to the probability of planar heme conformation, (**b**) I_1170_/I_1371_ ratio corresponds to the probability of the asymmetric vibrations of pyrrole half-rings, (**c**) I_1126_/I_1371_ ratio corresponds to the probability of the vibrations of the heme CH_3_ side radicals. Data are presented as mean values and error of the mean, ^#^ *p* < 0.05, ^##^ *p* < 0.01 for Cyt *c* mutants compared to Cyt *c* (WT), ** *p* < 0.01 for various Cyt *c* mutants compared to each other (nonparametric Mann–Whitney test), *n* = 5.

Similar spectral characteristics were calculated for oxidized Cyt *c* and its mutants (Figure 5). Among all the oxidized mutants, only Cyt *c* (K25E) demonstrates the increased probability of the planar heme (Figure 5a), and both mutants Cyt c (K25E and K25E/K72E) have a lower probability of the asymmetric pyrrol rings vibrations (Figure 5b) compared to Cyt *c* WT.

**Figure 6:**
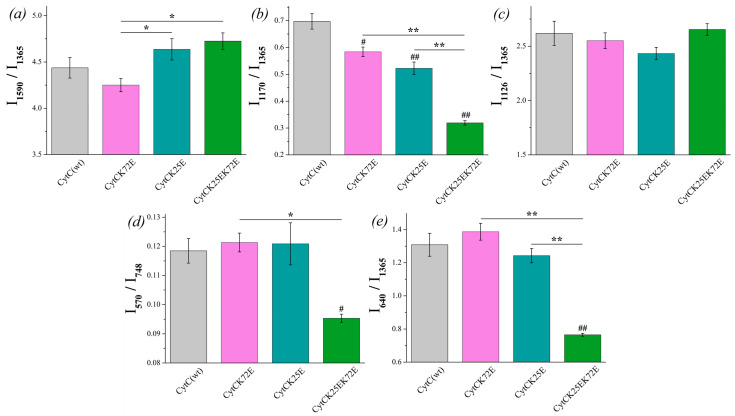
The ratios of the selected peaks intensities calculated from the RRS spectra of reduced Cyt *c* (WT) (light gray) and its mutants: Cyt *c* K72E (purple), Cyt *c* K25E (dark cyan), Cyt *c* K25E/K72E (green). (**a**) I_1590_/I_1365_ ratio corresponds to the probability of planar heme conformation, (**b**) I_1170_/I_1365_ ratio corresponds to the probability of the asymmetric vibrations of pyrrole half-rings, (**c**) I_1126_/I_1365_ ratio corresponds to the probability of the vibrations of the heme CH_3_-side radicals, (**d**) I_570_/I_748_ ratio corresponds to the probability of ruffled heme conformation, (**e**) I_640_/I_1365_ rate corresponds to the mobility of C-S bonds between heme and protein Cys-residues. Data are presented as mean values and error of the mean, ^#^ *p* < 0.05, ^##^ *p* < 0.01 for Cyt *c* mutants compared to Cyt *c* (WT), * *p* < 0.05, ** *p* < 0.01 for various Cyt *c* mutants compared to each other (nonparametric Mann–Whitney test), *n* = 5.

There is no significant difference between the I_1590_/I_1365_ ratio of reduced Cyt *c* mutants compared to Cyt *c* (WT), although the ratio I_1590_/I_1365_ demonstrates the tendency for the increase for Cyt *c* (K25E) and Cyt *c* (K25E/K72E) compared to WT (Figure 6a). The probability of planar conformation for reduced Cyt *c* (K25E) and Cyt *c* (K25E/K72E) is also significantly higher than that for the Cyt *c* (K72E) mutant. The relative contribution of asymmetric vibrations of pyrrole half-rings to symmetric ones (ratio I_1170_/I_1365_) is significantly lower for all Cyt *c* mutants compared to the wild type (Figure 6b). No difference was observed in the mobility of the heme CH_3_-side between Cyt *c* WT and mutants nor between the mutants themselves (Figure 6c). We also observed the significant decrease in the ratios I_570_/I_748_ and I_640_/I_1365_ for Cyt *c* (K25E/K72E) compared to WT (Figure 6d,e). These changes indicate the decrease in the probability of the ruffled heme conformation (that additionally confirms the tendency to the increase in the I_1590_/I_1365_ ratio) and the decrease in the mobility in the region of the C-S bond—the bond between the heme and Cys residue.

## 4. Discussion

Mutants of Ngb and Cyt *c* with amino acid substitutions in the contact surface do not show any frequency shifts of the characteristic peaks on the RRS and SERS spectra compared to the positions of these peaks on the spectra of the Ngb and Cyt *c* (WT). This is consistent with the fact that these substitutions do not lead to the dramatic conformational changes of the heme ring. Therefore, the studied mutant variants can be used to elucidate the role of individual amino acid residues of Ngb and Cyt *c* during Ngb–Cyt *c* transient complex formation.

The spectral characteristics of Ngb (E87K) are the closest to the ones of Ngb (WT) among all Ngb mutants, demonstrating the similarity of heme conformations in Ngb (E87K) and WT. This result can be explained by the fact that the Ngb Glu87 residue is located further from the heme compared to Glu60, Lys67 and Lys95 residues and, therefore, the amino acid substitution in the E87 location affects heme conformation to a smaller degree [3]. Reduced Ngb (E87K) heme also significantly differs from other Ngb mutants. Compared to these reduced mutants, Ngb (E87K) heme conformation is more planar, and the mobility of pyrrols rings is higher.

According to our results, Ngb (K95E) heme had undergone the greatest conformational changes in both oxidized and reduced states. The Ngb Lys95 residue is located close to the heme next to the proximal histidine (His96), which coordinates heme iron [3]. In an oxidized state, the heme environmental rigidity of Ngb (K95E) is significantly higher than that of Ngb (WT), Ngb (E60K) and Ngb (E87K), while the conformational mobility of the protein environment is significantly lower compared to the same Ngb species. For reduced Ngb mutants, these tendencies intensify, and Ngb (K95E) shows significantly higher heme environmental rigidity compared to all the other Ngb mutants and wild type.

Ngb (E60K) and the double-mutant Ngb (E60K/E87K) take similar spectral ratios in the reduced state. This is probably mainly due to E60K substitution, since Ngb Lys60 is linked to heme via water molecules [3], while E87K substitution does not contribute to the observed changes. Both mutants show a quite low probability of heme planar conformation and higher heme environmental rigidity in comparison with Ngb (WT). In the oxidized state, the probability of plane conformation for them is also decreased, although the heme environmental properties do not change compared to Ngb (WT).

It should be noted that the obtained results for Ngb mutants are in full agreement with our previous data on Ngb mutants’ absorption spectral properties in the UV-visible region [35]. The Soret bands in the UV-vis absorption spectra of Ngb E60K and K95E mutants are characterized by small shifts (1–3 nm) (Figure S2a,b). In addition, the spectra of the reduced forms of Ngb E60K and E60K/E87K are characterized by a well-defined Q-peak (582 nm). The aforementioned spectral features may be due to a change in the spin state of heme iron and a change in the electrostatic field near the heme [46,47].

To conclude, we suggest that among the Ngb mutants, the disruption of interaction with Cyt *c* is less probable for Ngb (E87K), where Glu87 presumably interacts with Cyt *c* residues Lys72 and Lys25. The most probable changes in interaction with Cyt *c* are those for Ngb (K95E), whereas Lys95 is supposed to interact with residues Ile81 and Gln16 of Cyt *c* [29,32,33].

The probability of planar heme conformation in the oxidized state is significantly higher for the mutant Cyt *c* (K25E) compared to the wild type and other Cyt *c* mutants, so we suppose that this Cyt *c* mutant can demonstrate the difference in the interaction with Ngb compared to Cyt *c* WT.

A reduced heme of double-mutant Cyt *c* (K25E/K72E) with substitutions in the interaction surface with Ngb is characterized by the highest probability of the planar conformation comparing to other mutants and Cyt *c* (WT). Notably, Cyt *c* (K72E) with only one substitution demonstrates no difference with Cyt *c* (WT) in heme conformation in both redox states, while double mutations (K25E/K72E) in reduced Cyt *c* result in the increase in the probability of the planar heme conformation and the decrease in the mobility of pyrrol rings and C-S bonds. It should be noted that the UV-vis absorption spectra of Cyt *c* mutants do not differ from WT Cyt *c* (Figure S2c,d).

## 5. Conclusions

The electron transfer is assumed to occur between reduced Ngb and oxidized Cyt *c* in the process of their reaction due to the electrostatic interaction between their contact sites and the optimal orientation of hemes *b* and *c*. By introducing the amino acid substitutions to the Ngb and Cyt *c* putative contact sites, we aimed to check whether they affect the heme conformation. We have found that reduced Ngb (E87K), having the mutation further from the heme than in other mutants, has a heme conformation similar to Ngb WT. Other studied reduced Ngb mutants demonstrate changed heme conformation with the lower probability of the heme planar conformation compared to WT. This change can affect Ngb heme orientation toward Cyt *c* heme and therefore influence the rate of the electron transfer. All oxidized Cyt *c* mutant molecules demonstrate the decrease in the probability of methyl-radicals vibrations, indicating the higher rigidity of the protein microenvironment. This change can affect the shift of the heme in its cleft under the interaction with Ngb influencing the rate of the electron acceptance. This work is the first stage of a large-scale study that will help to elucidate the mechanism of the Cyt *c* interaction with Ngb.

## Figures and Tables

**Table 1:** The mutagenesis primers for the introduction of amino acid substitutions in the putative interaction surface of Ngb and Cyt *c*.

Mutation	Oligonucleotide Sequence (5′→3′)
Ngb (E60K)	GTCTCTCCAGTCCA**A**AGTTTCTGGATCACATTC
Ngb (E87K)	GTCGAGCTTGGAA**A**AGTATCTTGCGAGTC
Ngb (K67E)	CTGGATCACATTCGC**G**AAGTGATGCTTGTG
Ngb (K95E)	CGAGTCTGGGTCGC**G**AACATCGTGCAGTTGG
Cyt *c* (K25E)	GTCGAAAAAGGTGGT**G**AGCACAAGACTGGTC
Cyt *c* (K72E)	CTTGGAAAACCCA**G**AGAAGTACATTCCTGGTAC

The changed codons are underlined, and the replacement nucleotides are printed in bold.

## Data Availability

All raw data are available from corresponding authors under reasonable request.

## Supplementary Materials

The following supporting information can be downloaded at https://www.mdpi.com/article/10.3390/cimb46040211/s1, Figure S1: Electrophoretic analysis in 12% SDS-PAGE of Ngb and Cyt *c* mutant variants; Figure S2: UV-vis absorption spectra of the (a) oxidized and (b) reduced forms of WT Ngb and its mutant variants and (c) oxidized and (d) reduced forms of WT Cyt *c* and its mutant variants.

## Abbreviations

Apaf-1—apoptotic protease activating factor-1; Cyt *c*—cytochrome *c*; ET—electron transfer; Ngb—neuroglobin; RRS—resonance Raman spectroscopy; SERS—surface-enhanced Raman spectroscopy; WT—wild type.
