# Supplementary material for: Charged Amino Acid Substitutions Affect Conformation of Neuroglobin and Cytochrome c Heme Groups
Source: Curr Issues Mol Biol. 2024 Apr 14;46(4):3364–78. doi: 10.3390/cimb46040211 (PMC11049214; doi:10.3390/cimb46040211)
Supplement: Supplementary file 1 [file cimb-46-00211-s001.zip › cimb-2922122-supplementary.pdf]

current issues in molecular biology

MDPI

Supplementary Materials

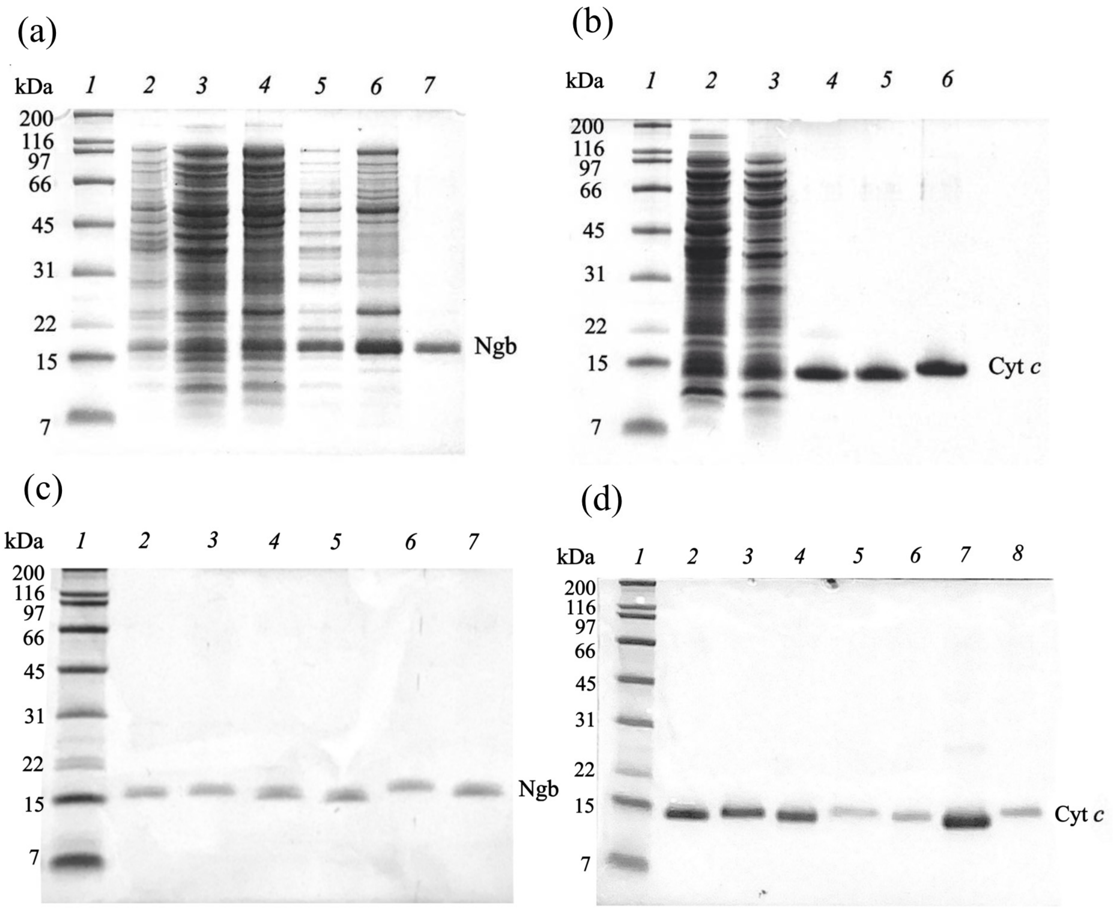

Figure S1. Electrophoretic analysis in 12% SDS-PAGE (Tris–Tricine buffer system) of the stages of isolation and purification of mutant (a) Ngb E60K/E87K: (1) molecular weight standard 7–200 kDa (Bio-Rad, USA); (2) total protein of cells of the producer strain at the end of expression; (3) soluble fraction of cell lysate after homogenization; (4) resuspended total protein after salting out; (5) combined fractions with Ngb after anion exchange chromatography; (6) combined fractions with Ngb after repeated salting out; and (7) purified protein preparation after gel filtration; (b) Cyt *c* K72E: (1) molecular weight standard 7–200 kDa (Bio-Rad, USA); (2) total protein of cells of the producer strain at the end of expression; (3) soluble fraction of cell lysate after homogenization; (4) combined fractions with Cyt *c* after cation exchange chromatography; (5) purified protein preparation after adsorption chromatography; (6) Cyt *c* WT; (c) purified Ngb preparations: (1) molecular weight standard 7–200 kDa (Bio-Rad, USA); (2) Ngb E60K; (3) Ngb E87K; (4) Ngb K67E; (5) Ngb K95E; (6) Ngb E60K/E87K; (7) Ngb WT; (d) purified Cyt *c* preparations: (1) molecular weight standard 7–200 kDa (Bio-Rad, USA); (2) Cyt *c* K25E; (3) Cyt *c* K72E; (4) Cyt *c* K25E/K72E; (5-7) Cyt *c* mutants that are not included in this study (M2, M4, 8Mut); (8) Cyt *c* WT

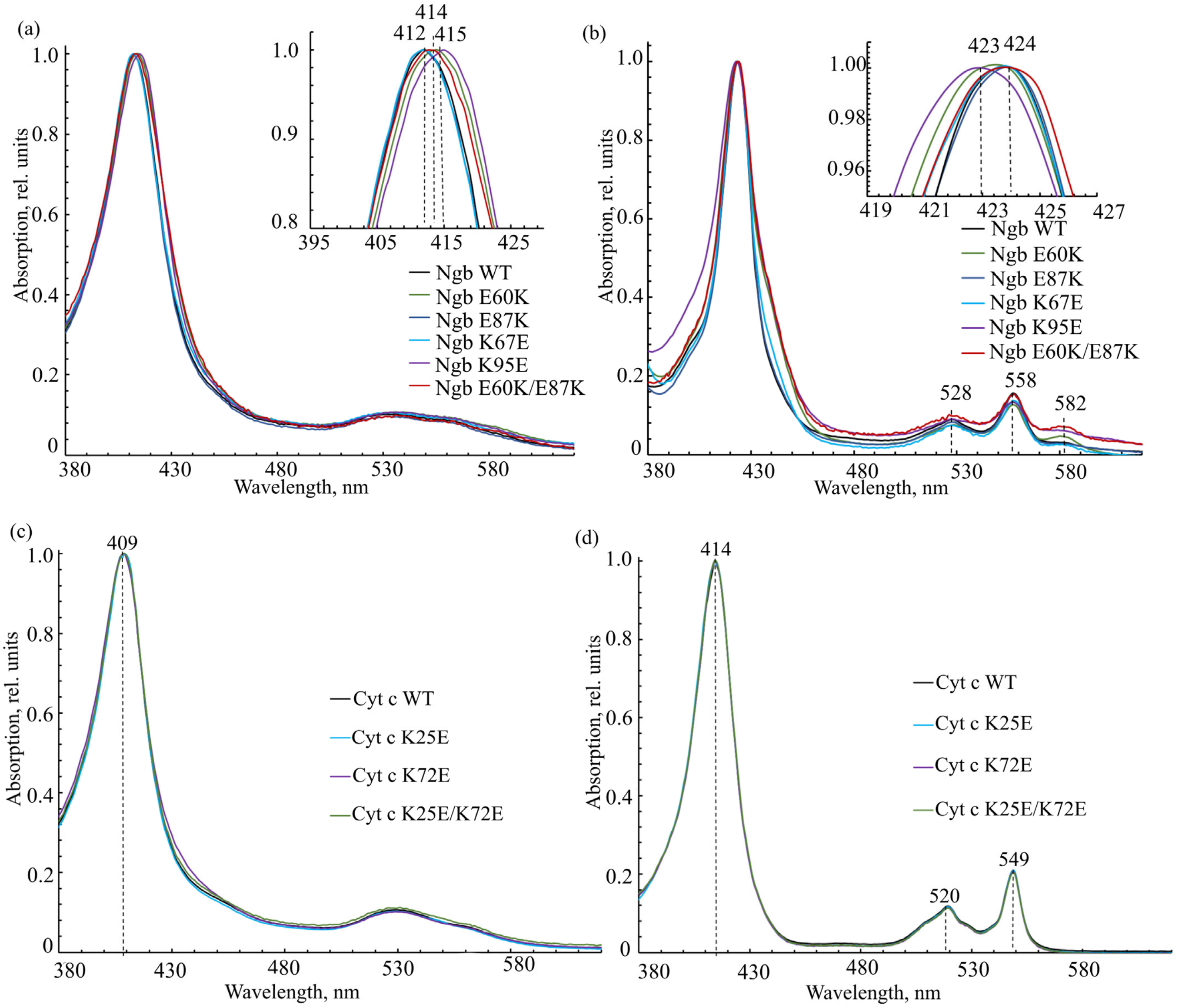

Figure S2. UV-vis absorption spectra of the (a) oxidized and (b) reduced forms of WT Ngb and its mutant variants and (c) oxidized and (d) reduced forms of WT Cyt *c* and its mutant variants
